# Heart Rate Variability in Healthy Non-Concussed Youth Athletes: Exploring the Effect of Age, Sex, and Concussion-Like Symptoms

**DOI:** 10.3389/fneur.2017.00753

**Published:** 2018-01-18

**Authors:** Melissa Paniccia, Lee Verweel, Scott Thomas, Tim Taha, Michelle Keightley, Katherine E. Wilson, Nick Reed

**Affiliations:** ^1^Rehabilitation Sciences Institute, University of Toronto, Toronto, ON, Canada; ^2^Concussion Centre, Bloorview Research Institute, Holland Bloorview Kids Rehabilitation Hospital, Toronto, ON, Canada; ^3^Faculty of Kinesiology and Physical Education, University of Toronto, Toronto, ON, Canada; ^4^Faculty of Medicine, Rehabilitation Sciences Institute, University of Toronto, Toronto, ON, Canada; ^5^Bloorview Research Institute, Holland Bloorview Kids Rehabilitation Hospital, Toronto, ON, Canada; ^6^Department of Occupational Science and Occupational Therapy, University of Toronto, Toronto, ON, Canada

**Keywords:** youth athlete, healthy, non-injured, heart rate variability, concussion, symptoms

## Abstract

**Background:**

Heart rate variability (HRV) is a non-invasive neurophysiological measure of autonomic nervous system regulation emerging in concussion research. To date, most concussion studies have focused on the university-aged athlete with no research examining healthy active youths. Corroborating changes in HRV alongside traditional subjective self-report measures (concussion symptoms) in the non-concussed state provides a foundation for interpreting change following concussion. The objectives were to (1) explore the influence of age and sex on HRV and (2) examine the relationship between HRV and baseline/pre-injury concussion symptom domains (physical, cognitive, emotional, and fatigue) in healthy youth athletes.

**Method:**

Healthy, youth athletes 13–18 years of age [*N* = 294, female = 166 (56.5%), male = 128 (43.5%)] participated in this cross-sectional study. Age, sex, and concussion-like symptoms were collected as part of a baseline/pre-injury assessment. The Post-Concussion Symptom Inventory-SR13 (PCSI-SR13) was used to collect domain scores for physical, cognitive, emotional, and fatigue symptoms. HRV was collected for 24 h. HRV measures included time (SDNN, RMSSD, and pNN50) and frequency (HF, HFnu, LF, LFnu, and total power) domain HRV measures. Variables were logarithmically transformed to increase robustness of linear regression models.

**Results:**

Older youth participants displayed significantly higher HRV compared to younger participants (*p* < 0.05). Females displayed significantly lower HRV compared to males (*p* < 0.05). A significant interaction effect between concussion-like symptoms and HRV indicated differential patterns as a function of sex (*p* < 0.05). Youth athletes who reported more cognitive symptoms had lower HRV (*p* < 0.05).

**Conclusion:**

HRV was found to have a significant relationship with a traditional clinical measure (subjective self-report of concussion-like symptoms) utilized in concussion assessment and management. Baseline/pre-concussion trends in HRV were significantly associated with age and sex, highlighting the value in understanding key demographic factors within the context of concussion-like symptoms.

## Introduction

The study of concussion in youth athletes has received increasingly more attention over the past decade, even more so since the recent Berlin Concussion Consensus Statement highlighted their focus on pediatrics (1). Internationally, it is estimated that four million children and youth present to the emergency department with a concussion annually (2, 3). A concussion is a form of traumatic brain injury induced by biomechanical forces; this can be caused by a direct blow to the head, or elsewhere on the body generating force to the head (4). Emerging research has highlighted the potential of exploring change in physiological measures following concussion, in the context of a multimodal approach (5, 6). However, a foundational first step in examining physiological measures in youth athletes following concussion is to understand natural variations present in a healthy population, alongside traditionally used clinical measures.

The cardiovascular system is predominantly controlled by autonomic regulation through the activity of sympathetic and parasympathetic branches of the autonomic nervous system (ANS). The cardiovascular control areas exist within the brainstem, through sympathetic and parasympathetic nerves (7), which work together to create sympathovagal balance or homeostasis. The balanced interplay between these two branches ultimately results in variations of beat-to-beat time intervals, in response to a variety of physical, environmental, and mental factors. Heart rate variability (HRV) is the non-invasive quantification of these beat-to-beat variations. Reduced HRV represents an attenuation of the autonomic regulatory capacity to support flexible adjustments in response to the environment, whereas increased HRV is reflective of good overall health and ability to respond to stressors (8).

Healthy ANS functioning within youth athletes has not yet been characterized and knowing this information could significantly enhance our understanding of post-injury comparisons and the variety of factors, demographic, and concussion-related that drive change in HRV. There have been numerous adult population-based studies on healthy HRV values stratified by age, sex, medication, and physical activity (9–12). However, unique developmental changes and pediatric milestones preclude these findings from being extrapolated to youth (13).

While several studies have explored the normal variation in ANS function across infants, children, and adolescents (14–18), little is known about how these trends are reflected within an athlete-specific population. Athletes, compared to non-athletes, have been described as having increased ANS plasticity, influenced by the changing environmental conditions present in sport participation, which ultimately result in an improved ability to be flexible and adaptable (19). For youth athletes who have been involved in sport from a young age, long-term physical training likely results in increased HRV (7). These findings have also been observed across various sport backgrounds such as cycling, canoeing, roller-skating, and volleyball (20–23). Thus, a first step in understanding this relationship is the examination of HRV in a healthy population.

The ANS of athletic youth appears to be different from non-athletic youth and as such, the factors of age and sex should be uniquely considered. Regarding age effects, a cross-sectional investigation of developmental trends of HRV from pre-school to adolescence, parasympathetic signal values (i.e., HF), values did not show change up to puberty, then decreased through adolescence [14–22 years old; (24)]. However, due to the dearth of literature that specifically examines the youth/adolescent population, a secondary aim of this study is to investigate the role that age plays on HRV in youth athletes. Regarding potential sex differences, a recent meta-analysis examining HRV sex differences in adults revealed that females have a smaller mean RR interval (higher HR) and lower HRV, which is particularly observed with long-term recordings (25). However, of the 172 studies included in the meta-analysis, only 7 studies focused on the adolescent population (10–20 years old), highlighting the scarcity of literature in this area. Across various studies, adolescent males consistently demonstrated higher values across SDNN, RMSSD, pNN50, HF, and LF, compared to females (24, 26). It is unclear if these differences will be present within an athlete-specific sample, and this study aims to contribute to that exploration.

Within baseline/pre-injury testing paradigms, it is standard to obtain self-reported symptom scores in addition to other standardized measures such as neuropsychological assessments (4). In the event of a concussion, these results are used to make comparisons with pre-injury scores in guiding return-to-play decisions. In particular, the value and focus on subjective symptom reporting has recently been debated (27, 28). Many studies have reported the presence of concussion-like symptoms in healthy (non-concussed) individuals (29–32). For example, in healthy child and youth athletes, Hunt et al. (30) found that fatigue was reported by more than half of the participants, nervousness was reported by 32% of youth girls, and 25% of the entire youth sample reported headaches, drowsiness, and difficulty concentrating. Thus, it is evident that concussion-like symptoms are non-specific; they are likely influenced by a confluence of personal and psychological factors such as school course load, emotional distress, and sport seasonality (33). The objectives of this study are the following: (1) to explore the effect of age and sex on HRV in healthy youth athletes and (2) to evaluate the relationship between HRV and concussion-like symptoms in a non-concussed state, and more specifically across physical, cognitive, emotional, and fatigue domains.

## Materials and Methods

Ethics approval was received from the Holland Bloorview Research Ethics Board at Holland Bloorview Kids Rehabilitation Hospital. All participants and their parents provided informed assent and consent, respectively, prior to their participation in this study.

### Study Design

A cross-sectional study of healthy youth athletes was conducted in which pre-injury/baseline data were collected on demographic information (age, sex) and concussion-related factors (concussion-like symptoms, previous history of concussion).

### Participants

A convenience sample of 294 healthy youth athletes (ages 13–18 years old) was recruited from local community sport organizations. Exclusion criteria were the following: participants with developmental delay, neurological condition, symptomatic from previous concussion, and non-English speaking. While participants were permitted to have a concussion history, they had to be asymptomatic and be fully participating in school and sport at the time of study entry. Participant demographics can be found in Table 1.

**Table 1:** Participant demographics.

Variable	*N* (%)
Age, M (SD)	14.22 (1.21)
Sex	
Males	128 (43.5%)
Females	166 (56.5%)
History of concussion	
Yes	85 (29%)
1	57 (19.5%)
2–3	27 (9.2%)
4	1 (0.3%)
Level of competition	
Competitive	249 (84.6%)
House league	17 (5.7%)
Recreation	8 (2.7%)

### Procedure

Upon receiving their informed consent, all participants completed a baseline/pre-injury assessment. Age, sex, and concussion history (i.e., number of previous concussions) were collected using a demographic collection form administered by research personnel. Level of competition (e.g., representative, house-league, or recreational), and primary sport (defined as the sport they played for the longest duration and at the highest level of competition) was also collected. The Post-Concussion Symptom Inventory-SR13 (PCSI-SR13; herein referred to as PCSI) was used as a self-report symptom assessment scale designated for adolescents to assess post-concussion symptoms. The PCSI is a 22-item self-report questionnaire used for youth ages 13–18 years; it has a seven point rating scale from 0 to 6 (0 = “not a problem,” 3 = “somewhat of a problem,” and 6 = “severe problem”). Both total scores (sum of all symptom ratings) and domain scores were derived. Symptoms within each domain include, but are not limited to: physical domain (e.g., headache, dizziness, blurred vision); cognitive domain (e.g., trouble concentrating, confused, mentally foggy); emotional domain (e.g., sad, irritable, nervous); and fatigue domain (e.g., fatigue, drowsiness). The PCSI has been found to have good validity and reliability among these age groups (32). Internal consistency has been found to range from 0.79 to 0.93 for the subscales and 0.94 for the total symptom score (32). Following the paper-and-pencil data collection, a 24-h HRV recording was collected *via* the Polar RS800CX watch and chest strap (RS800cx; Polar Electro, Kemple, Finland). The Polar technology used within this study has been deemed a valid tool to collect heart rate data, especially with respect to longer term recordings in the context of individuals who are physically active (34–36). Research personnel suited each participant with a chest strap that was suitable to their torso circumference. The heart rate sensor was buttoned to the central strap and research personnel then applied a conductivity gel to the inside of the strap. Participants were instructed on how to clasp the strap in position and to place the sensor directly below their sternum for optimal recording. Each participant was assisted by research personnel to adjust the chest strap and ensure the sensor was placed on the sternum. Participants were then instructed to put on the watch and wear it at all times. When all the equipment was set in place accurately, participants were instructed to press the start button on their watch, to commence heart rate recording. Due to the group-based testing environment of this large baseline study, there was not a consistent start time across all individuals. Based on sport team availability and school schedules, the start time varied between morning hours on the weekend and evening hours on weekdays. In accordance with the task force (37), HRV was collected over 24 h during normal daily routine to ensure greater stability of HRV measures. All participants were instructed to carry out their usual daily activities, with the exception of removing the device (and putting it back on) if they went swimming or played a contact sport. Participants were not controlled in terms of the time they decided to sleep and wake-up. As well, due to the group setting of testing within a larger baseline study, collecting specific sleep and wake times from participants upon their return of heart rate equipment was challenging, in which the majority did not do this or the information was incomplete. To address this limitation, the variable “Max HRV total power” was created to capture the 5-min window frame that the highest HRV value occurred, with the hypothesis that it would reflect a “sleep period” within the 24-h recording [i.e., highest parasympathetic activity occurs during sleep (38)]. This variable was accounted for in the statistical analysis.

### Data Analysis

#### HRV Analysis

The parameters of HRV were obtained non-invasively by calculating the RR wave interval of the heart *via* the QRS complex. The Polar RS800CX technology used in this study had a sampling rate of 1,000 Hz, which has been documented as sufficient time resolution (7). Time- and frequency domain measures were used in this study. Time-domain variables were selected for analysis as they are easily computed and have been shown to be highly correlated with HF power, a measure of parasympathetic activity (7). Power spectral analysis, *via* fast Fourier transformation, was used to derive frequency domain variables. Set out by the task force (37), the frequency bandwidths are characterized: HF (0.15–0.4 Hz) and LF (0.04–0.15 Hz).

The RHRV program, *via* R statistical programming (R Core Team, 2013), was used to compute time and frequency domain variables. For purposes of signal processing, it is crucial that the signals are corrected for ectopic and missed beats (39, 40). The automatic removal of spurious points was performed by the FilterNIHR function (replaces beats by a mean combination of preceding and following beats), creating RR intervals appropriate for analyses. The filter was set to a minimum of 25 beats per minute and a maximum of 200 beats per minute. For computation of the spectral components of the RR interval, the *InterprolatedNIHR* function was employed (4 Hz). Window frames were 300 s, 50% overlap. Duration of the recording influences many HRV indices, thus recordings that were <14 h and were not continuous recordings were excluded (7). Table 2 provides a definition for the time and frequency domain measures used in this study. The authors warrant caution in the interpretation of LF findings as considerable controversy on the usefulness of this measure has been documented. For example, Goldstein et al. (41) stated that LF likely is not representative of sympathetic autonomic modulation. Similarly, Billman (42) highlighted LF’s exceedingly poor relationship to sympathetic nerve activation, resulting in challenges delineating the physiological basis of this measure. As such, it is challenging to interpret the mechanisms driving change in an already exploratory study.

**Table 2:** Definition of time and frequency domain heart rate variability variables.

Variable	Units	Definition
SDNN	Milliseconds (ms)	SD of all RR intervals
RMSSD	Milliseconds (ms)	Root mean square of successive differences between RR intervals
pNN50	Percentage (%)	Percentage of adjacent RR intervals that differ from each other by more than 50 ms
HF	Milliseconds squared (ms^2^)	Power in the high frequency range (0.15–0.4 Hz)
HFnu	Percentage (%)	HF is divided by the sum of (HF + LF)
LF	Milliseconds squared (ms^2^)	Power in the low-frequency range (0.04–0.15 Hz)
LFnu	Percentage (%)	LF is divided by the sum of (HF + LF)
Total power	Milliseconds squared (ms^2^)	The variance of all RR intervals

#### Statistical Analysis

Descriptive analyses (mean, SD, range, and percentiles) were generated for all HRV variables, and stratified by sex. Multiple regression analyses were performed to investigate the effects of age, sex, and concussion-like symptoms on HRV in healthy youth athletes. The independent variables were age, sex, and PCSI physical, cognitive, emotional, and fatigue domains. The dependent variables were time (i.e., SDNN, RMSSD, and pNN50) and frequency domain (i.e., HF, HFnu, LF, LFnu, and total power) measures of HRV. The “Max HRV Total Power” variable was also included in this analysis to account for the variability in the start/stop time. Visual inspection of the dependent variable (i.e., HRV) indicated values outside the acceptable range of skewness (between −1 and 1) and kurtosis (near the value of 3). Thus, logarithmic transformations were applied to these variables to enhance statistical inference from the models. These transformations are appropriate based on data skewness and is in line with the analysis approach in the field of HRV in youth (43). Here, all diagnostic tests were indicative of meeting all model assumptions; constant error variance, no significant outliers, outcome variable is linearly related to the predictors, the predictors are not linearly dependent and nor do they display multicollinearity. R statistical programming package (R Core Team, 2013) was used for all analyses. Level of statistical significance was set to *p* < 0.05.

## Results

The sample in this study consisted of 294 youth between the ages of 13–18 (M = 14.22, SD = 1.21) years old [female = 166 (56.5%), male = 128 (43.5%)]. The majority of athletes played their primary sport at a high level of competition; 84% representative; 5.7% house league; 2.7% recreation (7.4% did not report level of competition). In terms of sport distribution, 62.5% played hockey, and 18.7% played soccer. The remaining sports were composed of various individual (10.2%; e.g., running, swimming, and skiing) and group (4%; e.g., baseball, basketball, and volleyball) sports. Youth participants also reported their exposure to sports on a weekly basis (M = 3.57, SD = 1.15, min = 1, max = 10). Preliminary univariate analyses were run to examine preexisting relationships between all variables of interest [demographic and concussion-related factors (PCSI domains and concussion history)]. There were no significant age differences between males and females. The majority of the sample (71%) had no previous history of concussion. Neither sex nor age were significantly related to previous concussions or number of previous concussions. The majority of youth athletes (78.2%) endorsed at least one symptom on the PSCI; 42.5% endorsed physical symptoms, 37.8% endorsed cognitive symptoms, 61.9% endorsed fatigue symptoms. Finally, no significant differences were found across HRV variables when comparing participants with no history of concussion to participations with a concussion history. Participant demographics are presented in Table 1. Across the 294 participants, the average length of the heart rate recording was 21.46 h (SD = 5.78) and length of recording was not found to be significantly associated with the HRV variables of interest. However, of the total 294 participants within this study, 147 participants had heart rate recordings which encountered multiple and sporadic start and stop times. Based on methodology recommendations and the RHRV computing backdrop, these were not sound to use for analysis in variables that required a continuous time series (44). It is important to note here that the limitation in data computation only applied to certain HRV variables. As such, they were excluded from specific regression models but not excluded from the study at large. Regarding “Max HRV Time,” M = 3:02 a.m., SD = 1 h, 55 min, min = 1:46 a.m., max = 6:55 a.m. Descriptive statistics on HRV variables are presented in Tables 3 and 4 (stratified by sex). Please see Table 5 for the *N* according to HRV variable.

**Table 3:** Descriptive statistics of heart rate variability variables for males.

Variable	*N*	Mean (SD)	Range	25th percentile	50th percentile	75th percentile
SDNN (ms)	128	193.33 (58.87)	88.81–445.82	149.97	186.64	222.79
RMSSD (ms)	128	68.59 (32.37)	20.31–183.30	44.91	59.65	89.82
pNN50 (%)	128	26.20 (13.90)	1.93–56.39	14.86	24.31	37.31
HF (ms^2^)*	61	444.06 (284.63)	42.45–1,113.41	207.21	372.05	684.97
HFnu*	61	34.93 (10.4)	16.08–58.04	27.31	35.68	41.48
LF (ms^2^)*	61	762.90 (373.93)	186.74–2,027.14	494.15	743.98	942.56
LFnu*	61	65.07 (10.46)	41.95–83.92	58.52	64.32	72.69
Total power (ms^2^)*	61	3,314.24 (1,681.27)	781.00–10,310	2,096.95	3,020.03	4,096.08

*A smaller *N* based on the inability to analyze those participants (*N* = 147) who had sporadic and multiple start and stop times within the 24-h recording. According to rigorous analysis methodology, it is not sound to link multiple recordings within one larger recording for the purpose of deriving frequency domain variables (RHRV, R Core Programming, 2013).

**Table 4:** Descriptive statistics of heart rate variability variables for females.

Variable	*N*	Mean (SD)	Range	25th percentile	50th percentile	75th percentile
SDNN (ms)	166	177.91 (52.89)	77.16–502.95	145.26	170.40	200.42
RMSSD (ms)	166	65.65 (28.58)	21.87–156.93	44.68	61.20	78.33
pNN50 (%)	166	25.28 (12.32)	0.93–54.68	15.99	24.51	32.84
HF (ms^2^)*	86	307.91 (306.49)	61.64–2,324.95	175.08	283.48	495.08
HFnu*	86	35.02 (8.61)	14.38–58.07	29.89	33.67	40.05
LF (ms^2^)*	86	624.52 (310.59)	173.77–1,905.63	364.48	590.09	779.69
LFnu*	86	64.98 (8.61)	41.93–85.62	59.94	66.33	70.10
Total power (ms^2^)*	86	2,574.99 (1,660.58)	944.00–7,305	1,611.71	2,455.76	3,272.76

*A smaller *N* based on the inability to analyze those participants (*N* = 147) who had sporadic and multiple start and stop times within the 24-h recording. According to rigorous analysis methodology, it is not sound to link multiple recordings within one larger recording for the purpose of deriving frequency domain variables (RHRV, R Core Programming, 2013).

**Table 5:** Linear regression model estimates of demographic and concussion-related factors.

	Estimate (SE)						
Heart rate variability variable	Sex	Age	PCSI physical	PCSI cognitive	PCSI emotional	PCSI fatigue	Previous concussion
log (SDNN) (*N* = 294)	−0.071 (0.03)*	0.031 (0.01)*	−0.013 (0.01)	−0.021 (0.009)*	0.015 (0.01)	0.015 (0.01)	0.061 (0.04)
RMSSD (*N* = 294)	−2.746 (3.60)	−0.133 (1.46)	−0.991 (1.13)	−2.195 (1.00)*	1.429 (1.43)	1.097 (1.58)	6.585 (3.90)
pNN50^a^ (*N* = 294)	−2.676 (1.85)	–	–	–	–	–	2.997 (1.67)
HF (*N* = 147)	−153.73 (64.71)*	−5.98 (20.33)	−31.41 (17.38)	−11.31 (14.99)	−17.77 (23.89)	4.57 (23.74)	43.47 (55.09)
HFnu (*N* = 147)	0.123 (1.62)	−0.731 (0.65)	0.001 (0.56)	−0.581 (0.48)	−0.217 (0.76)	1.018 (0.56)	2.394 (1.78)
log (LF) (*N* = 147)	−2.172 (1.09)*	0.247 (0.44)	−0.621 (0.38)	−0.049 (0.33)	0.137 (0.51)	0.325 (0.38)	0.471 (1.2)
LFnu (*N* = 147)	−0.123 (1.62)	0.731 (0.65)	−0.001 (0.56)	0.581 (0.48)	0.216 (0.76)	−1.018 (0.56)	−2.394 (1.78)
log (Total power) (*N* = 147)	−0.203 (0.08)*	0.017 (0.03)	−0.035 (0.03)	−0.021 (0.02)	0.018 (0.04)	0.037 (0.03)	0.110 (0.09)

*A significant p-value < 0.05.

*^a^The uniqueness of the pNN50 model. Level of fit indicated a removal of age as a factor and removal of each PCSI domain. Rather, PSCI total score enhanced the fit of the model and is described within the results section*.

### Max HRV Total Power

A linear regression was performed on Max HRV total power. Demographic factors (age and sex) were not significant in this model. Concussion-like factors (physical, cognitive, emotional, and fatigue domains; concussion history) were also not significant in this model.

### SDNN

**Figure 1:**
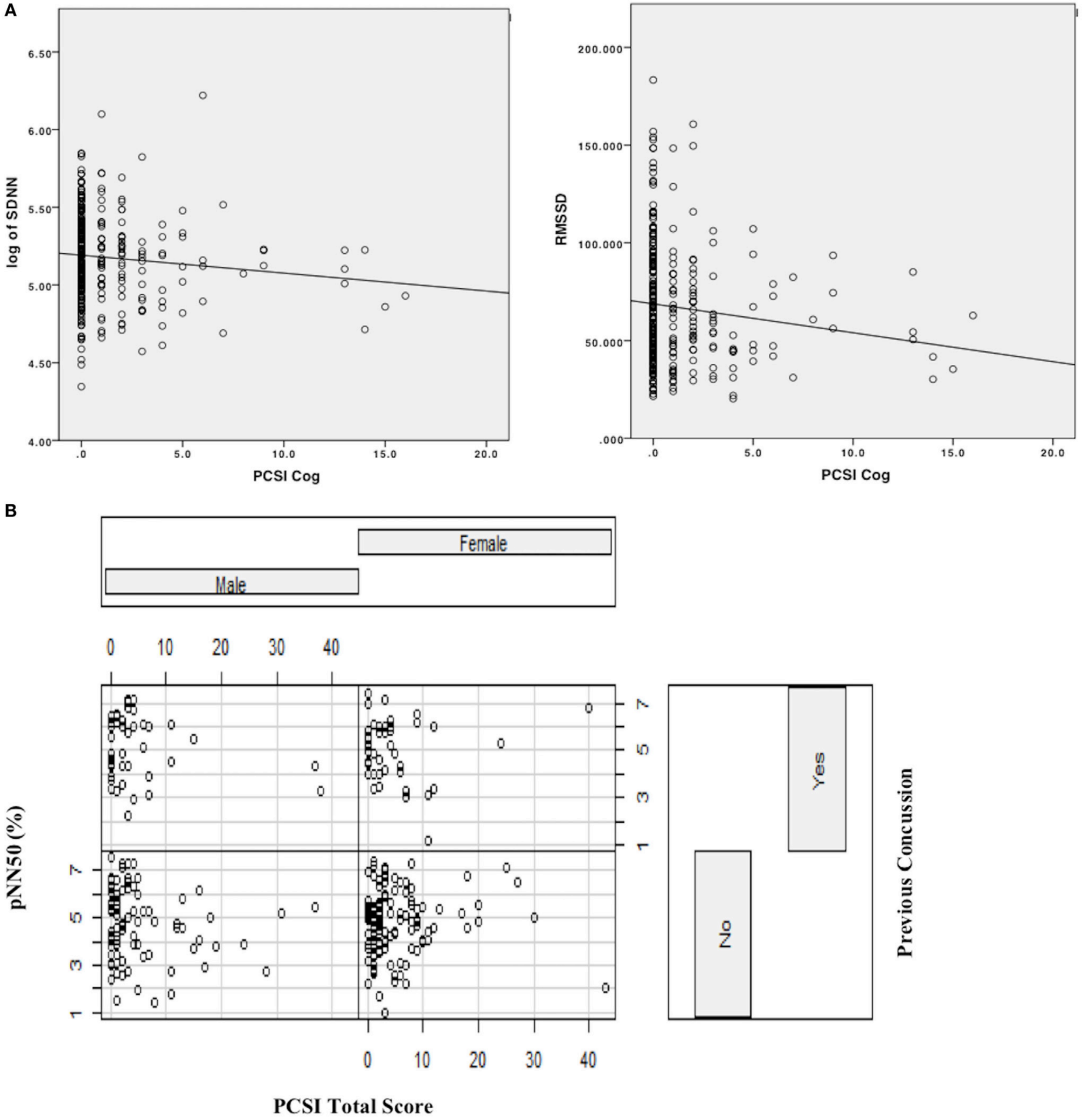
**(A)** Scatterplot with linear line of best fit depicting relationship between cognitive domain symptoms on PCSI and time-domain HRV variables (log [SDNN], RMSSD). **(B)** Interaction effect plot, depicting the relationship between PCSI total score by previous history of concussion as a function of sex (male/female) on pNN50.

Sex had a significant main effect, whereby females had lower SDNN compared to males (*B* = −0.071, SE = 0.033, *p* = 0.03). Age was also significant (*B* = 0.031, SE = 0.013, *p* = 0.023) in predicting SDNN, whereby older participants had higher SDNN compared to younger participants. Regarding concussion-like symptoms, the cognitive domain was the only domain demonstrating a significant effect (*B* = −0.021, SE = 0.009, *p* = 0.02). Here, there was a negative relationship, whereby youth who endorsed more cognitive symptoms had lower SDNN (Figure 1). Physical, emotional, and fatigue domains as well as previous concussion history were not significant in this model (Table 5).

### RMSSD

Age and sex did not have significant main effects within this model. Concussion-like symptoms in the cognitive domain had a main effect on RMSSD (*B* = −2.195, SE = 1.00, *p* = 0.03). A negative relationship was present, whereby youth who reported more cognitive symptoms had lower RMSSD (Figure 1). Physical, emotional, and fatigue domains as well as previous concussion history were not significant in this model (Table 5).

### pNN50

This was modeled differently from the above measures and a best-fit model was derived from including an interaction term and examining the PCSI total score (rather than the domain-specific variables). Age and sex did not have a significant effect on pNN50. The notable finding in this model was a significant interaction effect between PCSI total score and pNN50 as a function of sex (*B* = 0.424, SE = 0.211, *p* = 0.04). Here, the relationship between PCSI total score and sex was present in those who did not have a previous history of concussion (Figure 1). In females with no previous history of concussion, there was a positive association between PCSI total score and pNN50, whereby increased concussion-like symptom reporting was associated with increased pNN50. The opposite was found in males with no previous history of concussion, whereby increased concussion-like symptom reporting was associated with decreased pNN50 (Table 5).

### HF

While age did not have a significant effect, sex did have a main effect on HF (*B* = −153.73, SE = 64.71, *p* = 0.019), whereby females had lower HF compared to males. The physical, cognitive, emotional, and fatigue domains of the PCSI as well as previous concussion history were not significant in this model (Table 5).

### HFnu

Demographic factors such as age and sex did not have a significant effect on HFnu. The physical, cognitive, emotional, and fatigue domains of the PCSI as well as previous concussion history were not significant in this model (Table 5).

### LF

While age did not have a significant effect, sex did have a main effect on HF (*B* = −2.172, SE = 1.09, *p* = 0.04), whereby females had lower LF compared to males. The physical, cognitive, emotional, and fatigue domains of the PCSI as well as previous concussion history were not significant in this model (Table 5).

### LFnu

Demographic factors such as age and sex did not have a significant effect on HFnu. The physical, cognitive, emotional, and fatigue domains of the PCSI as well as previous concussion history were not significant in this model (Table 5).

### Total Power

Sex had a significant main effect on total power (*B* = −0.203, SE = 0.08, *p* = 0.01), whereby females had lower total power compared to males. Age, concussion-like domains, and previous history of concussion were not significant in this model (Table 5).

## Discussion

The purpose of this study was to examine the effect of development, sex, and concussion-like symptoms on a measure of cardiac autonomic function (i.e., HRV) in a sample of healthy youth athletes. This area of research has been limited by: (1) the lack of focus on the youth population, compared to studies on children or adults; (2) the specific exploration of demographic trends within an athlete population; and (3) the corroboration of a novel physiological measure with traditionally used clinical measures. To the author’s knowledge, this study is one of the first to examine HRV in a large sample of healthy youth athletes, in the context of a traditional clinical tool commonly used in the assessment and management of concussion (i.e., subjective symptom reporting). This study contributes new knowledge in the realm of understanding the physiological correlates associated with everyday, non-specific, concussion-like symptoms. This study found significant effects of sex and age across HRV measures as well as the impact of everyday cognitive symptoms on HRV. Previous history of concussion, while accounted for, did not appear to markedly effect HRV across all measures.

### Age Effects

This study found an age effect on HRV in one (SDNN) of the five HRV variables examined, whereby older youth participants had higher SDNN compared to younger youth athletes. While age-related trends in HRV are likely related to age-related changes in heart rate (45), these developmental trends were not found in other measures of HRV. However, when comparing mean HRV values to those found in a systematic review comparing athletic versus non-athletic adults, the youth values found in this study were all markedly higher (SDNN, RMSSD, and HF) than those reported in the review (46). Fukuba et al. (47) explored HRV characteristics of pre- and post-adolescent Japanese individuals between the ages of 8 and 20 years and found no effect of age on parasympathetic and sympathetic indices. It is possible that cardiac autonomic modulation may develop until approximately 7–8 years old and stabilize throughout adolescence (47). This mechanism has been emphasized as a stabilization of sympathovagal balance, noted by increased cholingeric and decreased adrenergic modulation of HRV (18). Seifert et al. (48) found significant age trends of HRV in the group of children under the age of 11 years old (i.e., HRV decreased with increasing age). However, these were not present in the group over the age of 11 years old (48). Given that (1) the sample in this study was composed of 13- to 18-year-old athletes and (2) 17- to 18-year olds only made up 5.1% of the total sample, the high performance athletic status, and age range may not have been sensitive enough to capture developmental change. It is important to note that this finding was only present in SDNN, compared to other HRV variables within this study; here, it is challenging to decipher if SDNN is truly impacted by age or if this reflects a chance finding. A more rigorous exploration of development within this age range of youth athletes is warranted.

### Sex Effects

This study found consistent sex differences across both time (i.e., SDNN) and frequency domain HRV measures (i.e., HF, LF, and total power), whereby females displayed decreased HRV compared to males. These results are mirrored in a recent meta-analysis on sex differences in HRV across the life span, which found that females have less variability in the time-domain measures, lower total power, yet higher HF power compared to males, which is contrary to the findings of this study (25). Our findings are consistent with adolescent literature, which have shown that boys displayed higher HRV in measures that reflect parasympathetic activity [SDNN, RMSSD, pNN50, and HF; (49–51)]. These results are not surprising given that girls typically experience puberty 2 years prior to boys (52). Here, pre-pubertal hormonal changes such as estrogen and progesterone as well as substantial changes of hormonal levels during induction of ovulation play a role in driving change in HRV [i.e., lowering HRV; (53)] Thus, the timing of pubertal development may coincide with the emergence and maturation of neural autonomic mechanisms (13, 26, 54). Sex differences found in this study may also be linked to athleticism; boys in this age range may engage in more physical training thus increasing their HRV compared to girls (55). While there were no significant differences between males and females in the level of sport exposure within this study (males: M = 3.45, SD = 1.04; females: M = 3.66, SD = 1.22), it is unclear how the level of intensity and duration of sport exposure differed. Given that baseline sex differences in HRV exist in healthy youth, it can be hypothesized that ANS vulnerabilities, which occur during this developmental stage, may potentially explain the role that sex plays in concussion recovery where it has been found that females report more concussions (56), experience a greater severity of symptoms and a protracted recovery compared to males (57). Future research on how these sex differences manifest following a concussion is warranted.

### Concussion-Like Symptoms

Across a multitude of studies (30–32), it has become evident that there may be significant overlap in daily variations of concussion-like symptoms with those experienced by an injured population. Here, it is important to note that due to the fact that concussion symptoms are non-specific, the interest in this study was to conceptualize concussion-like symptoms in the context of everyday variability (i.e., experienced by healthy, non-injured youth). Within the context of sustaining a concussion, however, subjective symptom reporting is problematic in athletic populations who may be prone to inaccurate reporting due to fear of a prolonged return-to-play process (58). This study found that endorsing cognitive symptoms (while healthy) was reflected in lower HRV. Cortico-subcortical networks, which regulate harmony between the sympathetic and parasympathetic branches of the ANS, structurally and functionally link to cognitively related processes (8). Within the domain of cognitive symptoms experienced day-to-day, reported difficulties with memory, concentration, confusion, and slower mental processing can feedback to the ANS as a form of physiological stress, causing changes in cardiovascular regulation. In a recent meta-analysis on the effects of acute mental stress in healthy adults revealed significantly lower HRV in those who experience more stress on tasks of computer work, arithmetic, and academic examination (59), authors stated a potential autonomic balance shift toward sympathetic activation and parasympathetic withdrawal. While it is not surprising that high-performing youth athletes experience variations in concussion-like symptoms (30), it is important to consider the potential post-concussion implications of these findings. Clinically, baseline/pre-injury trends in cognitive symptoms and HRV can contextualize and inform comparisons post-concussion. It may be that youth athletes who report high levels of cognitive symptoms and display decreased HRV at baseline have a protracted recovery in the event of a concussion compared to those who do not experience cognitive symptoms on a daily basis. This study provides a foundation to that inquiry and demonstrates the presence of a physiological signal in daily forms of cognitive stress.

Endorsing symptoms in physical, emotional, and fatigue domains did not reveal any effects in this study. In non-athlete populations of healthy adolescents, a scoping review revealed relationships between daily emotional symptoms of anxiousness, nervousness, and sadness and decreased HRV; it is hypothesized that the ability to regulate emotions relates to ANS flexibility and adaptability in the face of life stressors (60). However, being a healthy youth athlete may be a protective factor in these concussion-like domains. For example, youth athletes, compared to non-athletes have been shown to have better psychosocial functioning, emotional well-being, self-efficacy, and psychological resilience (61). It may be plausible that daily physical symptoms are mitigated by sport participation as well. However, it is worth noting that the PCSI is not a comprehensive assessment on each of these domains and expansion of these domains within targeted clinical assessments is warranted within this population.

Finally, the interaction between total symptom score on HRV as a function of sex is noteworthy; in youth athletes with no history of concussion, females who reported more total concussion-like symptoms displayed increased HRV, whereas males who reported more total symptoms displayed decreased HRV. This finding is in line with concussion research which revealed a suppression of LF power in males, associated with mood disturbances, even following concussion symptom resolution (62). These sex differences may also be explained by psychophysiological theories which state that in the context of stress, females may be more likely to “tend-and-befriend,” seeking out a support network and resources for coping (63). It is worth noting that the expectation of change in HRV, regardless of sex, would be that an increase in concussion-like symptoms is associated with a decrease in HRV. However, given that this study specifically examined a healthy population, it may be that females’ ability to self-regulate, seek out resources, and/or recognize daily stress feeds back positively to the ANS in ways that are uniquely different from the ways healthy males react to stress. Afferent ANS pathways have demonstrated the release of oxytocin *via* oxytocin neurons to buffer stress and serves to reduce the impact of stressful events; this feedback loop has been implicated in inducing bradycardia (lowered heart rate) which in turn increases HRV (64). Conversely, males may employ the “fight-or-flight” reaction in response to stress, increasing sympathetic activity and decreased HRV (63). While the ability to cope with concussion-like symptoms was not captured in this study, the constructs of self-management and self-regulation would be key to consider in future research.

### Limitations

This study is not without limitations. First, the HRV methodology employed a 24-h recording, which was taken during normal everyday conditions. While long-term recording approach has been deemed ecologically valid (65), the major limitation of this study was the processing issue that occurred as a result of sporadic start and stop times unintentionally driven by participants. Here, the ability to compute frequency domain variables was limited, resulting in a significantly lower *N* for those analyses. Thus, collecting HRV data over 24 h may not be feasible with this age group. Future research using “ecologically valid” recordings should employ more control in monitoring the parameters of collecting data. For example, Bornas et al. (65) collected HRV data over a duration of 2.5 h, whereby all youth participants had a consistent classroom schedule. Here, youth were able to freely go about their morning classroom routine, the environment acting as a consistent control across all participants. Interpreting change in HRV can also be limited by other factors such as frequency and intensity of physical activity. Associations of HRV have been described with physical activity in which increased participation in physical activity increased HRV (66–68). On the other hand, a previous study using controlled conditions (69) revealed that the variance of the average heart rate was also large across age groups; this finding was also replicated in a review on heart rate in adolescents (45). Thus, it is unclear if the large variance reflects the differences of the individual characteristics of cardiovascular control rather than the differences of activity. Restrictions on physical activity in this study were not feasible or appropriate for long-term recordings, given the diverse activity repertoires of youth (school, sport, extracurricular activity). However, given that the sample was predominantly representative/high-performing athletes (i.e., likely engage in moderate to vigorous forms of physical activity on a regular basis), there is some homogeneity in this sample. The limitation in not accounting for physical activity does mirror the current state of literature (25) and future exploration could include the concurrent collection of HRV and actigraphy technology to capture the type, frequency, and intensity of physical activity.

Second, development was assumed to be captured by age; while these two variables are highly correlated, more rigorous evaluation such as Tanner’s stage (70) may have yielded a more sensitive means to capture developmental trends in ANS function (26). This recommendation is likely useful given the variation in autonomic modulation occurring after puberty (71). However, due to the fact that youth athletes were in the presence of other youth during the baseline/pre-injury testing protocol, it was not feasible to collect highly sensitive information without the risk of disclosing private information. Furthermore, a potentially confounding effect may have been menstruation cycle in females. Studies investigating the effects of the menstrual cycle on cardiac autonomic function have stated that regulation of autonomic tone is modified during the menstrual cycle, in which the alteration of ovarian hormones might be responsible for changes seen in cardiac autonomic activity (72, 73). Based on the age range of this sample (13–18 years old), it is unclear how the menstrual cycle may or may not have played a role in symptom reporting or in HRV changes. Future study capturing stage of development in addition to time period of menstrual cycle would likely contribute to a more robust understanding of developmental variations in ANS function.

It is worth nothing that the complexity of cardiac autonomic regulation was not comprehensively captured within this study. As mentioned, HRV is influenced by an array of different physiological inputs to the ANS. This study examined linear methods of HRV analysis and thus, the findings in this study can only be applied to linear measures of HRV. However, the use of non-linear methods of analysis (e.g., DFA and SampEn) is warranted to construct a broader understanding on healthy ANS function in youth athletes; it is possible that non-linear methods reveal sensitivities in self-reported symptoms that linear methods cannot capture (65, 74, 75). Finally, the use ECG lead-based technology may have yielded enhanced accuracy in recording the heart rate signal compared to the Polar technology used within this study (76).

## Conclusion

Findings indicate the presence of variability in reporting concussion-like symptoms in a healthy, non-concussed sample of youth athletes. Here, youth athletes who reported more cognitive symptoms had lower HRV. This finding is a foundational first step in exploring a novel physiological measure in the context of a traditional, subjective measure (PCSI) used in the clinical assessment of youth athletes. Sex differences were consistent across HRV measures revealing that females have lower HRV compared to males. Interestingly, the relationship between concussion-like symptom reporting and HRV differed between males and females (with no previous concussion), and assessments of coping and self-management may provide additional information at elucidating this difference.

## Ethics Statement

This study was carried out in accordance with the recommendations of “Tri-Council Policy Statement: Ethical Conduct for Research Involving Humans (TCPS 2), Holland Bloorview Research Ethics Board” with written informed consent from all subjects. All subjects gave written informed consent in accordance with the Declaration of Helsinki. The protocol was approved by the “Holland Bloorview Research Ethics Board.”

## Author Contributions

MP conceptualized the study objectives, data collection and analysis, and drafted manuscript; LV contributed to the conceptualization of the study with particular expertise in methodology and data processing, contributed to literature review and manuscript draft; ST participated in the methodology and data processing design and editing manuscript; TT participated in the conceptual design with expertise in data processing, editing manuscript; MK conceptualized study objectives and edited manuscript; KW participated in data collection, literature review, and editing manuscript; NR is the principal investigator on this manuscript with a broader role in supervising the proposal, data collection and analysis, methodology, diligent manuscript draft review, and editing for submission.

## Conflict of Interest Statement

This research was conducted in the absence of any commercial or financial relationships that could be construed as a potential conflict of interest.
